# Novel, male-produced aggregation pheromone of the cerambycid beetle *Rosalia alpina*, a priority species of European conservation concern

**DOI:** 10.1371/journal.pone.0183279

**Published:** 2017-08-21

**Authors:** Alenka Žunič Kosi, Yunfan Zou, Michal Hoskovec, Al Vrezec, Nataša Stritih, Jocelyn G. Millar

**Affiliations:** 1 National Institute of Biology, Department of Organisms and Ecosystem Research, Ljubljana, Slovenia; 2 University of California, Department of Entomology, Riverside, California, United States of America; 3 Institute of Organic Chemistry and Biochemistry ASCR, Prague, Czech Republic; Montana State University Bozeman, UNITED STATES

## Abstract

Several recent studies have demonstrated the great potential for exploiting semiochemicals in ecology and conservation studies. The cerambycid beetle *Rosalia alpina* represents one of the flagship species of saproxylic insect biodiversity in Europe. In recent years its populations appear to have declined substantially, and its range has shrunk considerably as a result of forest management and urbanization. Here, we collected volatile chemicals released by males and females of *R*. *alpina*. Analyses of the resulting extracts revealed the presence of a single male-specific compound, identified as a novel alkylated pyrone structure. In field bioassays in Slovenia, traps baited with the synthesized pyrone captured both sexes of *R*. *alpina*, indicating that the pyrone functions as an aggregation pheromone. Our results represent the first example of a new structural class of pheromones within the Cerambycidae, and demonstrate that pheromone-baited traps can provide a useful tool for sampling *R*. *alpina*. This tool could be particularly useful in the ongoing development of conservation strategies for the iconic but endangered Alpine longicorn.

## Introduction

Semiochemicals have been used as effective tools for monitoring and control of populations of native and invasive pest insect species for many years (e.g., [1–5]), but to date, have been less frequently exploited for monitoring of nonpest species, such as endangered species which are targets for conservation efforts [6–8]. The implementation of the Directive on the Conservation of Natural Habitats and of Wild Fauna and Flora or Habitats [9] in Europe stimulated establishment of monitoring schemes for target species of European conservation concern, among which are a number of rare and endangered insects. These endangered species, and species with elusive or cryptic life styles, are often difficult to monitor due to ineffective survey procedures [10]. In at least some cases, survey procedures might be greatly improved by employing volatile pheromones or other semiochemical attractants [11]. In recent years, considerable progress has been made in identifying pheromones of endangered species (e.g., [12–21]), but to date, pheromone-based detection and sampling have only been used to monitor a few endangered coleopteran and lepidopteran species [6, 22–31], probably due to the lack of sufficient knowledge about the communication systems of insects of concern for conservation.

**Fig 1:**
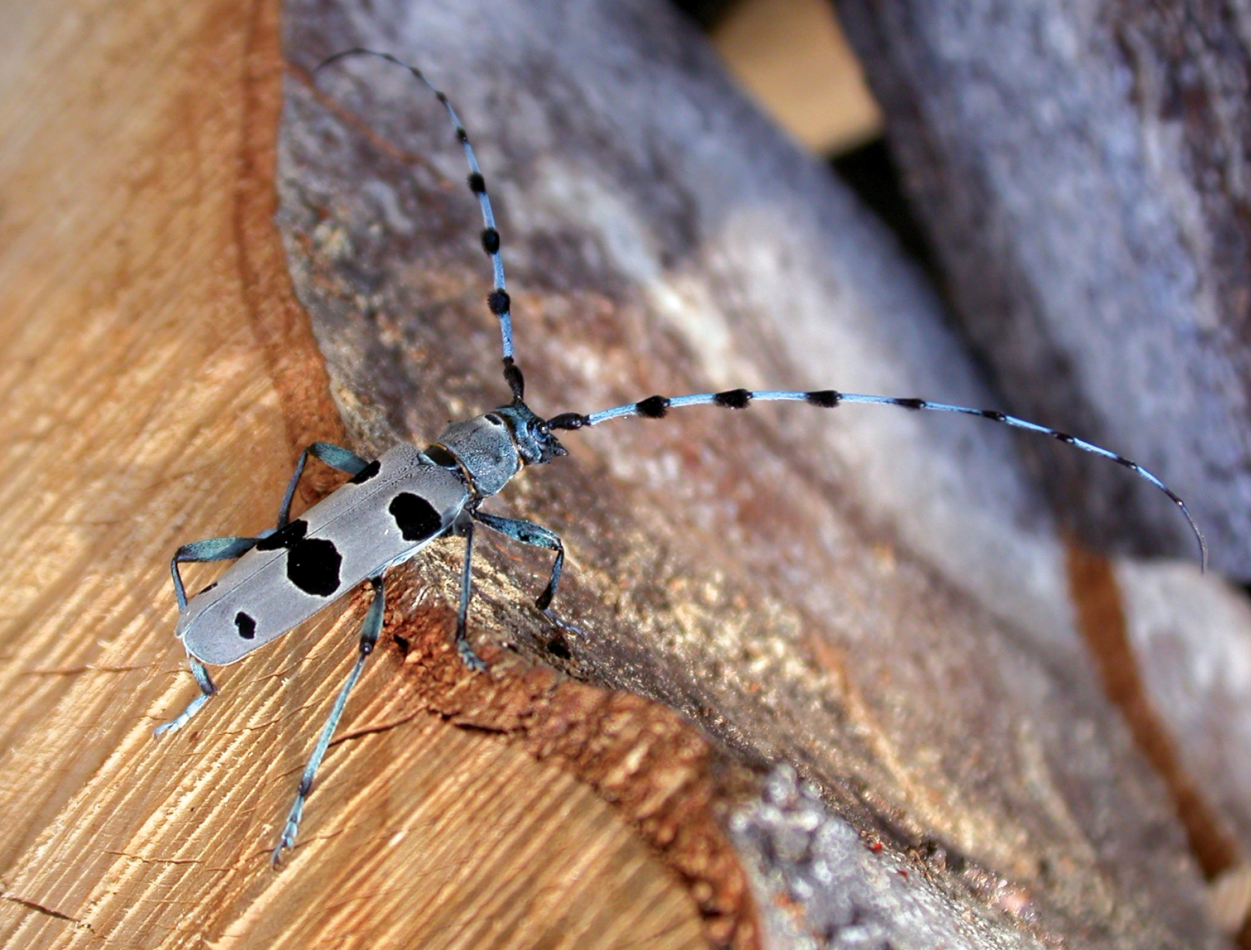
Male of Alpine longicorn (*Rosalia alpina*) sitting on the freshly cut beech (*F*. *sylvatica*) timber.

Saproxylic beetles are among the most threatened animal groups in Europe, owing to decline of their habitat due to intensive forest management, the decline of old-growth forests, and the decreasing amount of deadwood in forest stands [32, 33]. The Alpine longicorn (*Rosalia alpina* L., Coleoptera: Cerambycidae) (Fig 1) is among the most charismatic [34, 35] and threatened obligate saproxylic beetles in Europe [36]. It is listed as a priority species for conservation under Annex II of the EU Habitats Directive [37], and classified as “vulnerable” on a global scale [38]. *Rosalia alpina* is mainly associated with old forest stands of the European beech (*Fagus sylvatica* L.), but has been found on other deciduous tree species (e.g., *Quercus*, *Ulmus*, *Carpinus*, *Fraxinus* [36, 39]). Its populations across Europe have greatly declined in recent years, and the species’ range has shrunk significantly, mainly because of degradation and loss of habitat due to modern forest management, and urbanization [40–42]. Its presence is restricted to patches of mature forest with significant amounts of the dead wood required for development of its larvae [43]. Because of the short imago life stage and the cryptic life history of its other life stages, its ecology, biology, and long-term population trends are not well known, although such information is crucial for development of effective conservation management. Current knowledge of its distribution has been assembled from historical records, occasional findings, and from surveys by visual inspection of fresh beech stumps and woody debris, individual trees, and cut timber, including counting emergence holes and larval galleries (e.g., [35, 40, 41, 44–47]). These rather haphazard methods of data collection and analysis are very labour-intensive, difficult to standardize, and are environmentally, temporally, and spatially biased [48, 49]. More resource-efficient and standardized methods for detecting the presence and abundance of this species would be of substantial value for on-going conservation and monitoring efforts in Europe.

Over the past decade, intensive research on the chemical ecology of longhorn beetles (Cerambycidae) has shown that volatile semiochemicals play an important role in their reproductive behaviours [50]. Field tests of cerambycid pheromones have demonstrated their potential for detection and management of cerambycid beetles as organisms of high economic and ecological importance in forest ecosystems [51, 52]. Volatile pheromones produced by either sex, often in combination with volatiles of the host plant, can attract mates over long distances. In addition, the cumulative data suggests that closely (e.g., congeners) and sometimes even more distantly related species (e.g., in the same tribe or subfamily), often share or have very similar pheromone components.

In the present study we report the identification, synthesis, and field bioassays of the major volatile compound produced sex-specifically by male *R*. *alpina*, and discuss its (dis)similarity to the pheromone of the closely related North American congener *Rosalia funebris* [53]. The active compound is not only a novel natural product, but also represents the first example of a new structural class of pheromones within the family Cerambycidae. We discuss the possibility of developing pheromone-based tools for detection and sampling of this iconic, European, IUCN's Red-List species.

## Materials and methods

### Collection of volatiles

Adult *R*. *alpina* of both sexes were collected between 26 and 28 July 2014 from a pile of freshly cut beech trunks at Mt. Boč in NE Slovenia (lat. 46°17', lon. 15°36', 600 m a.s.l.), a site where an abundant population of this species had been reported [54]. Beetles were collected under licence No. 35601-75/2012-8 issued by the Slovenian Environmental Agency.

In the laboratory of the National Institute of Biology (NIB), beetles were left under ambient room conditions for 24 h before collection of headspace odours. Volatiles were collected from males (N = 7) and females (N = 2) under ambient laboratory conditions (23 ± 1°C, and 65% RH ± 2°C, natural light conditions) between 28 and 30 July. Individual beetles were placed in modified 250 ml Ball® Mason-style canning jars that contained paper tissues (white, 4.4" x 8.4" Kimtech Science Precision Wipes, Kimberly-Clark, USA, moistened with distilled water), and a leafless twig of the European beech (*Fagus sylvatica*, [L.] (3 cm long, 0.5 cm wide). The jar lids were fitted with a Teflon® liner and two brass bulkhead unions (Swagelok®, San Diego Valve and Fitting Co., San Diego CA, USA), for attachment of inlet and outlet air lines. The inlet line was connected to a 2 cm diameter x 20 cm long copper tube filled with activated charcoal granules to clean the incoming air. The outlet line was fitted with a volatiles trap consisting of a glass tube (4 mm diameter and 30 mm long) containing a 10 mm long bed of thermally-desorbed activated charcoal (200 mesh; Fisher Scientific, Pittsburgh, PA, USA) held in place by Soxhlet-extracted (ether) glass wool plugs. The collection tube was then connected to a flow meter-controlled vacuum source. Charcoal-filtered air was pulled through the chamber and collector at ~ 200 ml/min. Aerations were run for 3 d, after which the collectors were extracted with dichloromethane (3 rinses, total volume of 500 μl). Extracts were stored in a refrigerator (2°C) until used for analyses.

### Analysis of insect-produced volatiles

Extract samples were initially analyzed at NIB by coupled gas chromatography-mass spectrometry (GC-MS) using an Agilent 6890N GC (Agilent, Santa Clara, CA, USA) coupled to a 5973 mass selective detector. The GC was fitted with a DB-23 column (60 m x 0.25 mm diameter, 0.15 μm film thickness; J&W Scientific, Folsom CA, USA). The GC was programmed from 40°C for 1 min, 10°C min^-1^ to 230°C, using helium carrier gas. Injections were made in the splitless mode, with an injector temperature of 250°C. Additional unit resolution GC-MS analyses were done at University of California, Riverside in splitless mode using an HP6890 GC coupled to an HP5973 mass selective detector (Hewlett-Packard, now Agilent, Santa Clara, CA, USA). The GC was fitted with a DB-17 column (30 m x 0.25 mm x 0.25 μm film; J&W Scientific), programmed from 40°C for 1 min, 10°C min^-1^ to 280°C, with helium carrier gas. Kovats retention indices were calculated relative to straight-chain hydrocarbons. The exact mass was determined with a Waters GCT instrument in electron impact ionization mode (70 eV).

An aliquot of extracts from male beetles was reduced by addition of ~1 mg 5% Pd on carbon, and stirring for 2 h under H_2_. After filtration through Celite, the resulting product was analysed by GC-MS. A second aliquot was concentrated just to dryness, and then treated with 200 μl of a solution of LiAlH_4_ in ether (5 mg/ml) for 2 h at room temp. The resulting mixture was diluted with 1 ml ether, then carefully quenched with 1 M aqueous HCl (foams!). The ether layer was washed with saturated NaHCO_3_ solution and brine, dried over anhydrous Na_2_SO_4_, concentrated, and analysed by GC-MS.

Two extracts were combined and concentrated to < 5 μl under a gentle stream of nitrogen, then 0.25 ml of deuterated methylene chloride was added and the sample was concentrated again to < 5 μl. This was repeated once more, concentrating down to ~10–15 μl, the solution then was transferred to a 1 mm microbore NMR tube, and the sample was run on a Bruker Avance 600 spectrometer at 600 MHz, taking ^1^H, COSY, NOESY, HSQC, and HMBC spectra.

### Synthesis of the male-produced compound (see Fig 2)

Tetrahydrofuran (THF) was dried by distillation from sodium/benzophenone ketyl under argon. Anhydrous MeOH was purchased from Aldrich Chemical (cat. No. 322415) and was used as received. Anhydrous DMF was purchased from Alfa Aesar (cat. No. 43997) and was used as received. All other solvents were obtained from Fisher Scientific (Pittsburgh PA, USA; Optima grade) and were used as received. Mass spectra were recorded as described above. ^1^H and ^13^C NMR spectra were recorded on a Varian INOVA-400 (400 and 100.5 MHz respectively) spectrometer (Palo Alto, CA), as CDCl_3_ solutions unless specified otherwise. ^1^H NMR chemical shifts are expressed in ppm relative to residual CHCl_3_ (7.27 ppm). ^13^C NMR chemical shifts are reported relative to CDCl_3_ (77.16 ppm). Reactions with air- or water-sensitive reagents were carried out in oven-dried glassware under argon. Crude products were purified by flash column chromatography or vacuum flash chromatography on silica gel.

**Fig 2:**
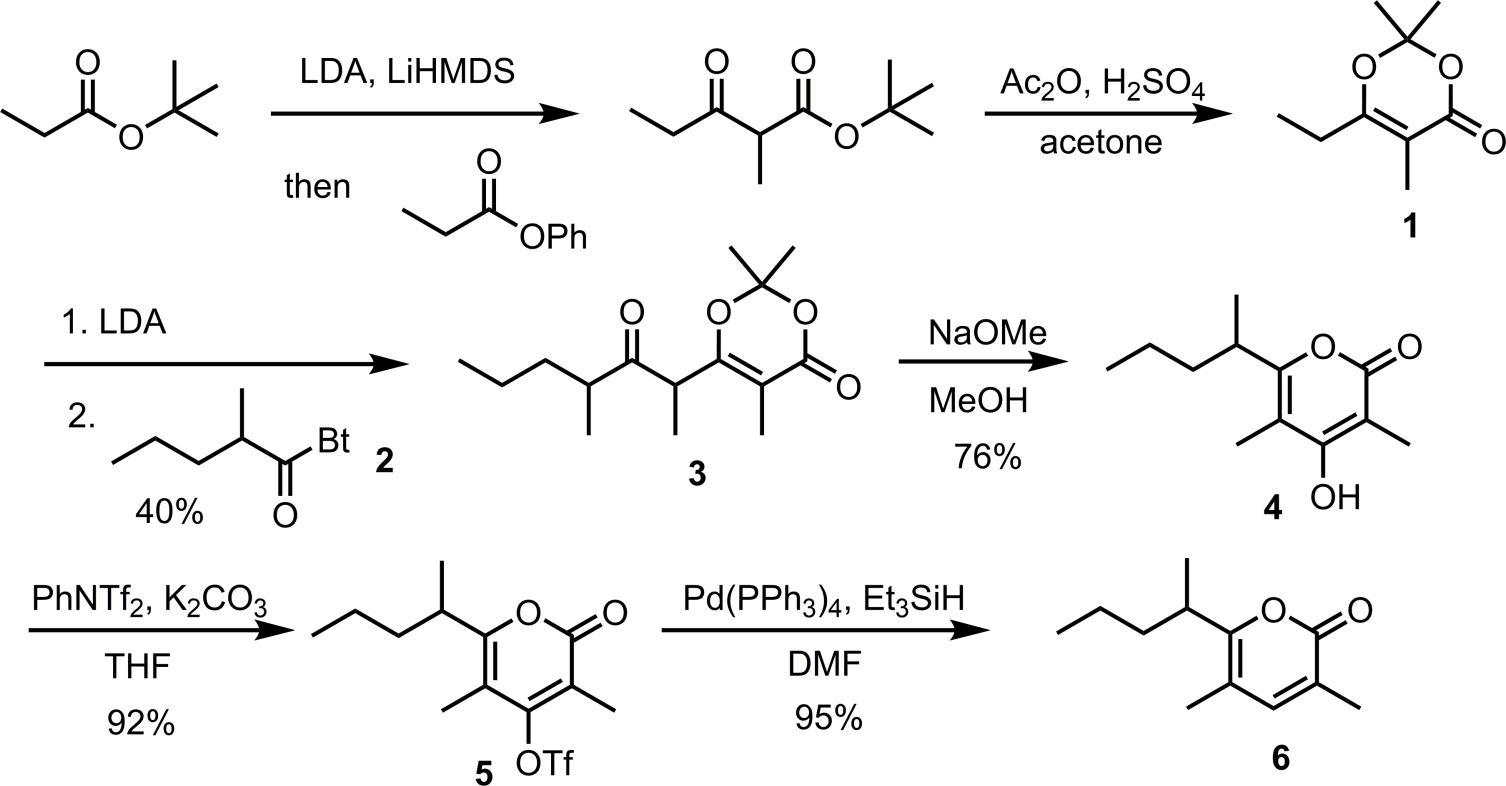
Synthesis of the racemic *Rosalia alpina* pheromone.

#### 1-Benzotriazol-1-yl 2-methylpentan-1-one (2)

2 was prepared according to the general procedure reported in Katritzky et al. [55]. Thus, SOCl_2_ (2.2 mL, 30 mmol) was added to a stirred solution of benzotriazole (14.30 g, 120 mmol) in CH_2_Cl_2_ (150 mL) at room temperature. After 0.5 h, 2-methylvaleric acid (3.8 mL, 30 mmol) was added and stirring was continued for 2 h. The white precipitate was filtered off and washed with CH_2_Cl_2_ (twice, 200 mL total). The combined organic solution was washed with aqueous 2N NaOH (thrice, 400 mL total), water, and brine, then dried over anhydrous Na_2_SO_4_. After evaporation of the solvent, the crude product was Kugelrohr distilled (120°C / 0.3 Torr) to give 2 as a colorless liquid (6.22 g, 95%). ^1^H NMR (CDCl_3_, 400 MHz) δ 8.30 (d, *J* = 8.0 Hz, 1H), 8.11 (d, *J* = 8.0 Hz, 1H), 7.64 (t, *J* = 8.0 Hz, 1H), 7.49 (t, *J* = 8.0 Hz, 1H), 4.12 (m, 1H), 1.95 (m, 1H), 1.65 (m, 1H), 1.37–1.51 (m, 2H), 1.40 (d, *J* = 7.2 Hz, 3H), 0.93 (t, *J* = 7.2 Hz, 3H); ^13^C NMR (CDCl_3_, 100.5 MHz) δ 178.60, 146.36, 131.39, 130.42, 126.18, 120.21, 114.75, 38.95, 35.95, 20.48, 17.35, 14.10; MS (*m*/*z*, rel. abundance) 41 (18), 43 (33), 63 (11), 64 (13), 71 (15), 90 (57), 91 (14), 118 (79), 119 (17), 146 (100), 147 (13), 175 (10), 217 (M^+^, 15).

#### 6-(1,3-Dimethyl-2-oxo-hexyl)-2,2,5-trimethyl-1,3-dioxin-4-one (3)

A solution of *n*-BuLi (2.24 M in hexanes, 1.6 mL, 3.6 mmol) was added dropwise to a solution of *i*-Pr_2_NH (0.50 mL, 3.6 mmol) in THF (5 mL) at -78°C. The reaction flask was transferred to an ice-water bath and stirring was continued for 15 min. The reaction flask was cooled to -78°C again, and a solution of 6-ethyl-2,2,5-trimethyl-1,3-dioxin-4-one 1 (0.51 g, 3.0 mmol, prepared as described by Zhang et al. 2015 [56] in THF (2 mL) was added dropwise. The reaction mixture was stirred for 1 h at -78°C, then 2 (0.98 g, 4.5 mmol) was added dropwise. The reaction mixture was stirred and allowed to warm to room temperature overnight. The mixture was quenched with saturated aqueous NH_4_Cl solution, and the aqueous phase was extracted with Et_2_O. The combined organic phase was washed with saturated aqueous Na_2_CO_3_ solution, water, and brine, then dried over anhydrous Na_2_SO_4_. After evaporation of the solvent, the crude product was Kugelrohr distilled (85°C / 0.5 torr) to remove most of the unreacted starting material 1 (1 and product 3 are very close on TLC, Hex/EtOAc = 9/1, 1 R_f_: 0.28, 3 R_f_: 0.22). The residue was purified by flash column chromatography to give 3 as a yellow liquid (0.32 g, 40%). ^1^H NMR (CDCl_3_, 400 MHz) δ 3.76 and 3.69 (q, *J* = 6.8 Hz, total 1H), 2.59–2.70 (m, 1H), 1.893 and 1.891 (s, total 3H), 1.48–1.70 (m, 7H), 1.14–1.36 (m, 6H), 1.01–1.08 (m, 3H), 0.87 and 0.84 (t, *J* = 7.2 Hz, total 3H); ^13^C NMR (CDCl_3_, 100.5 MHz) δ 209.95, 209.60, 163.09, 162.96, 162.49, 105.40, 102.00, 47.82, 46.71, 44.34, 44.30, 36.30, 34.75, 25.90, 25.72, 24.26, 24.08, 20.52, 20.45, 17.73, 16.34, 14.15, 14.13, 12.46, 12.33, 10.32; HRMS calcd. for C_15_H_24_O_4_ 268.1675, found 268.1733.

#### 4-Hydroxy-3,5-dimethyl-6-(1-methylbutyl)-pyran-2-one (4)

MeONa (1.13 g, 21.0 mmol) was added to a stirred solution of 3 (1.88 g, 7.0 mmol) in anhydrous MeOH (17.5 mL) at 0°C. The reaction mixture was allowed to warm to room temperature and stirred for 3 d. The reaction was quenched with 1M aqueous HCl, and the aqueous phase was extracted with CH_2_Cl_2_. The combined organic phase was washed with water and brine, and dried over anhydrous Na_2_SO_4_. After concentration, the crude product was purified by vacuum flash chromatography (Hex/EtOAc = 5/1 to 1/1) to give 4 as a white solid (1.11 g, 76%). ^1^H NMR (CDCl_3_, 400 MHz) δ 8.93 (br s, 1H), 2.85–2.94 (m, 1H), 2.01 (s, 3H), 1.98 (s, 3H), 1.59–1.68 (m, 1H), 1.38–1.46 (m, 1H), 1.17–1.27 (m, 2H), 1.14 (d, *J* = 6.8 Hz, 3H), 0.84 (t, *J* = 7.2 Hz, 3H); ^13^C NMR (CDCl_3_, 100.5 MHz) δ 167.36, 166.30, 161.99, 107.33, 98.35, 36.79, 34.38, 20.74, 18.47, 14.09, 9.80, 8.87; MS (*m*/*z*, rel. abundance) 43 (17), 55 (10), 83 (41), 111 (15), 112 (24), 139 (100), 140 (11), 155 (12), 167 (15), 210 (M^+^, 37); HRMS calcd. for C_12_H_18_O_3_ 210.1256, found 210.1289.

#### 3,5-Dimethyl-6-(1-methylbutyl)-2-oxo-2H-pyran-4-yl triflate (5)

A mixture of 4 (5.05 g, 24 mmol), K_2_CO_3_ (9.95 g, 72 mmol) and *N*-phenyl-bis(trifluoromethanesulfonimide) (9.00 g, 25.2 mmol) in anhydrous THF (96 mL) were stirred at 60°C for 4 h. The reaction mixture was cooled to room temperature, poured into water, and extracted with Et_2_O. The combined organic phase was washed with saturated NH_4_Cl, water, and brine, then dried over anhydrous Na_2_SO_4_. After concentration, the crude product was purified by flash chromatography (Hex/EtOAc = 95/5) to give 5 as a light yellow liquid (7.57 g, 92%). ^1^H NMR (CDCl_3_, 400 MHz) δ 2.86–2.95 (m, 1H), 2.12 (s, 3H), 2.03 (s, 3H), 1.64–1.74 (m, 1H), 1.46–1.55 (m, 1H), 1.16–1.32 (m, 2H), 1.21 (d, *J* = 6.8 Hz, 3H), 0.88 (t, *J* = 7.2 Hz, 3H); ^13^C NMR (CDCl_3_, 100.5 MHz) δ 163.73, 163.60, 156.77, 118.49 (q, *J*_CF_ = 318 Hz), 115.67, 106.59, 36.78, 35.11, 20.77, 18.32, 14.06, 11.58, 10.73; MS (*m*/*z*, rel. abundance) 41 (11), 43 (32), 55 (11), 69 (16), 71 (13), 83 (32), 111 (13), 138 (11), 139 (20), 153 (11), 181 (24), 215 (14), 244 (19), 271 (100), 272 (13), 299 (17), 342 (M^+^, 41); HRMS calcd. for C_13_H_17_F_3_O_5_S 342.0749, found 342.0831.

#### 3,5-Dimethyl-6-(1-methylbutyl)-pyran-2-one (6)

Pd(PPh_3_)_4_ (0.46 g, 0.40 mmol) and triethylsilane (2.5 mL, 16.0 mmol) were added sequentially to a solution of triflate 5 (2.74 g, 8.0 mmol) in anhydrous DMF (56 mL). The resulting mixture was heated at 60°C for 1 h. The solution became deep red and the reaction appeared complete by TLC. The reaction mixture was cooled to room temperature, poured into a mixture of water and Et_2_O, and the resulting mixture was filtered through Celite, rinsing with ether. The organic phase was separated and washed with water and brine, dried over anhydrous Na_2_SO_4_, concentrated, and the crude product was purified by flash chromatography (Hex/EtOAc = 9/1) to give 6 as a light yellow liquid (1.48 g, 95%). NMR spectra were taken in CD_2_Cl_2_ so that they could be directly compared with the spectra obtained from 6 isolated from *R*. *alpina*. ^1^H NMR (CD_2_Cl_2_, 400 MHz) δ 6.95 (d, *J* = 1.2 Hz, 1H), 2.75–2.84 (m, 1H), 1.99 (d, *J* = 1.6 Hz, 3H), 1.94 (s, 3H), 1.61–1.71 (m, 1H), 1.42–1.50 (m, 1H), 1.13–1.29 (m, 2H), 1.17 (d, *J* = 6.8 Hz, 3H), 0.88 (t, *J* = 7.2 Hz, 3H); ^13^C NMR (CD_2_Cl_2_, 100.5 MHz) δ 164.45, 162.11, 144.74, 122.45, 110.13, 37.36, 34.90, 21.23, 18.72, 16.58, 15.30, 14.37; MS (*m*/*z*, rel. abundance) 41 (12), 67 (23), 95 (11), 123 (100), 124 (11), 151 (24), 194 (M^+^, 30); HRMS calcd. for C_12_H_18_O_2_ 194.1307, found 194.1359.

### Field bioassays of the synthetic pheromone

Field research and collection of beetles were conducted in Slovenia under licence No. 35601-75/2012-8 issued by the Slovenian Environmental Agency, and part of the fieldwork was conducted within the scope of Monitoring Program of Natura 2000 beetle species in Slovenia. Field bioassays of the synthesized compound were conducted at three study sites, Mts. Boč, Kum, and Krim (Table 1), dominated by beech forests (*Fagus sylvatica* L.) with known *R*. *alpina* populations (of low- to high-density) [57]. At each site we set up 3 replicates of black flight-intercept panel traps (1.1 m high×0.3 m wide, modelled after cross-vane panel traps WitaPrall IntPt–Nassfalle, sold by Witasek PflanzenSchutz GmbH, Austria), each containing 3 treatment traps and a control trap. Lures consisted of low-density polyethylene press-seal bags (»Zipper» seal sample bag, 5× 7.5 cm, 2 μm wall thickness, Fisher Scientific, Pittsburgh, PA, USA). Treatment 1 consisted of lures loaded with 50 mg of the racemic pheromone in 0.45 ml of isopropanol; treatment 2 consisted of a pheromone lure and a lure loaded with host plant volatiles (1 ml (*Z*)-3-hexen-1-ol + 1 ml ethanol, both from Sigma-Aldrich, Steinheim, Germany); treatment 3 consisted of host plant volatiles alone; control lures were loaded with isopropanol. Lures were suspended in the central open area of each trap. Trap collection cups (white plastic, 8 cm diameter, 17 cm height) were filled with 200 ml of saturated aqueous NaCl solution to preserve captured beetles. Traps were positioned at least 20 m apart in transects, and were suspended from tree branches at a height of 1.5–2 m on randomly selected tree species (e.g., *Fagus*, *Abies*, *Quercus*, *Fraxinus*). The field bioassay was deployed for approximately 30 d after the first *R*. *alpina* adult was observed, corresponding to the estimated population peak in Slovenia from end of June to the beginning of August [58]. Treatments initially were assigned randomly to traps, and in subsequent trap checks (in total 5), lures were shifted one position along each transect to minimize position effects. Traps were checked for beetles once per week. Lures were replaced when completely dry.

**Table 1:** Study sites used in field bioassays of *Rosalia alpina* pheromone bioassays carried out in Slovenia.

Study site	Coordinates, altitude	Period	#replicates/ site	#trap days	Treatments
Boč	lat. 46°17'	21.6.–9.8.2016	3	43	1. pheromone compound (F)
	lon. 15°36'				2. pheromone compound +
	500–700 m a.s.l				host plant volatiles (F+HV)
					3. host plant volatiles (HV)
					4. control (C)
Kum	lat. 46°4'	24.6.– 2.8.2016	3	33	1. pheromone compound (F)
	lon. 15°8'				2. pheromone compound +
	680–820 m a.s.l.				host plant volatiles (F+HV)
					3. host plant volatiles (HV)
					4. control (C)
Krim	lat. 45°52'	24.6.– 8.8.2016	3	39	1. pheromone compound (F)
	lon. 14°23'				2. pheromone compound +
	450–600 m a.s.l.				host plant volatiles (F+HV)
					3. host plant volatiles (HV)
					4. control (C)

### Statistical analyses

In total we had samples from 43 trap days at Mt. Boč, 33 at Mt. Kum, and 39 trap days per trap at Mt. Krim. We tested the attractiveness of the *R*. *alpina* pheromone both using pooled data from all study sites, and using data from the individual study sites separately. For pooled data (blocked by sites and date), differences between treatments in the mean number of adults of both sexes captured per 10 trap days were tested with the Kruskal-Wallis nonparametric ANOVA [59, 60] (using Past Software Package) because the data were not normally distributed. Pairwise multiple comparisons of the four treatments were done using the nonparametric Mann-Whitney U test with Bonferroni adjustment [59, 60] (using PAST). Data were expressed as mean ± SE and differences were considered as statistically significant at P < 0.05. The sex ratio of captured beetles was compared to a nominal 1:1 ratio with the χ^2^ goodness-of-fit test (SPSS software). For the individual study sites, differences between treatments in the numbers of beetles captured/trap were tested with the nonparametric Friedman's test (blocked by trap check date and replicate) [60] (R software R-3.0.0). Pairwise multiple comparisons of the four treatments at each study site were done using the nonparametric Dunn’s test, with Bonferroni adjustment [61] (R software R-3.0.0).

## Results

### Identification of insect-produced volatiles

**Fig 3:**
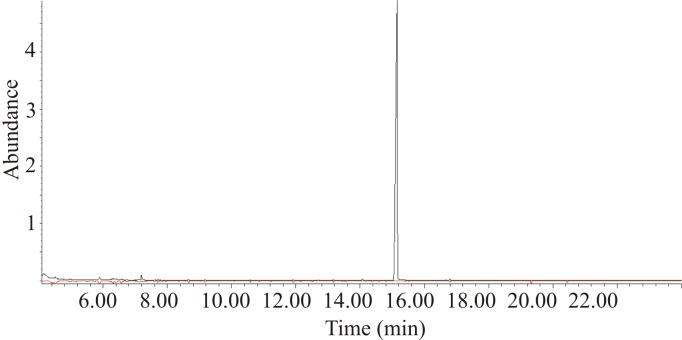
Representative GC-MS chromatograms of extracts of headspace volatiles from a male (upper trace) and female *Rosalia alpina* (lower, red inverted trace), showing the single abundant male-specific compound.

**Fig 4:**
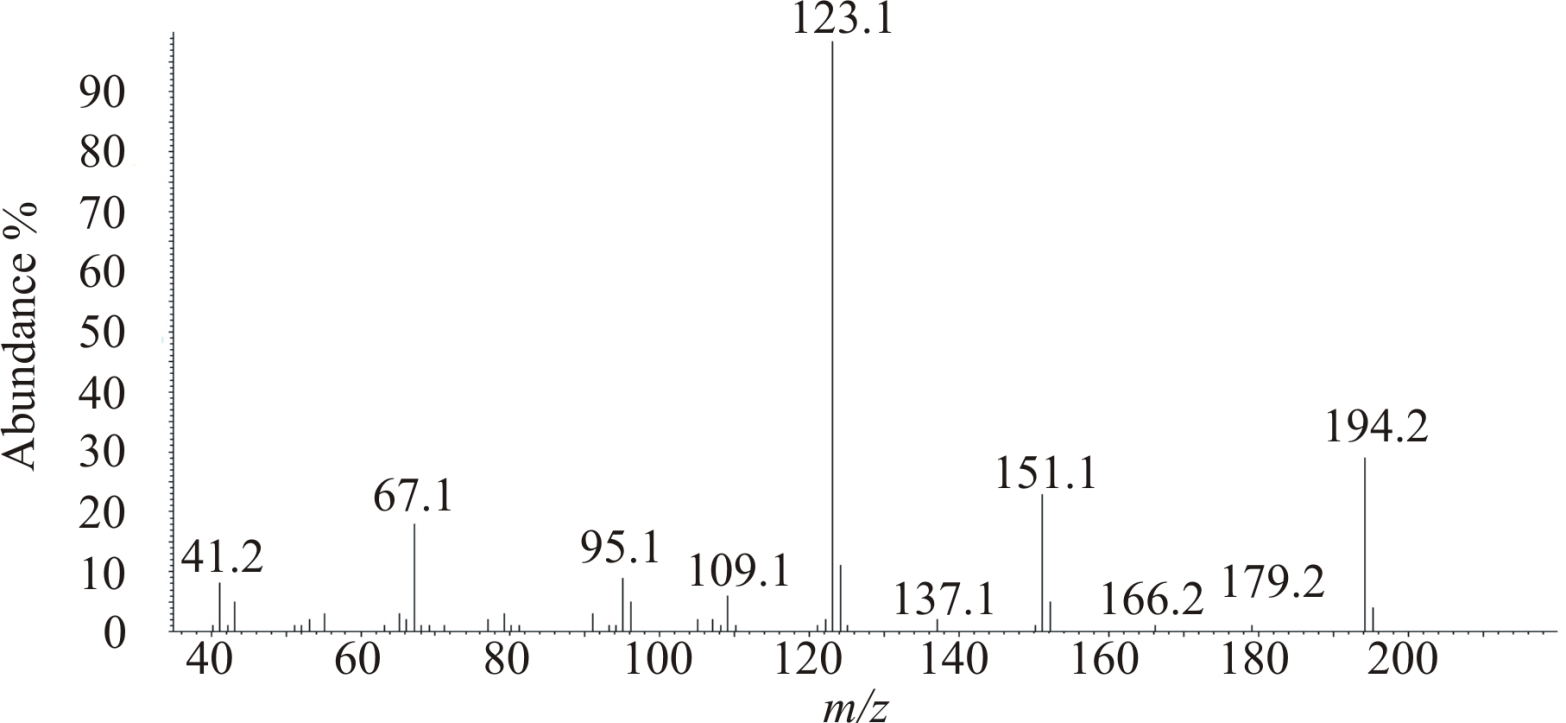
EI mass spectrum of male-specific compound from *Rosalia alpina*.

Analyses of extracts of headspace volatiles produced by both sexes by GC-MS revealed a single sex-specific peak in six out of seven extracts of males (Fig 3), with a strong molecular ion at *m/z* 194 (30% of base peak at *m/z* 123) (Fig 4). The spectrum showed relatively little fragmentation overall, suggestive of a cyclic or highly conjugated structure. The molecular formula was confirmed as C_12_H_18_O_2_ via the high resolution mass spectrum (calcd 194.1301; found 194.1302), corresponding to 4 sites of unsaturation. Pd-catalyzed hydrogenation produced two closely-eluting isomers, both of which exhibited a weak molecular ion at *m/z* 198 (1% of base peak at *m/z* 127), suggesting the presence of two C = C double bonds in the parent structure, and an unsaturated, alkylated ring, reduction of which had resulted in the two isomers. In addition, the base peak at *m/z* 127, from loss of 71 mass units from the reduced parent molecule, suggested the favoured loss of a 5-carbon alkyl chain from a relatively stable C_7_H_11_O_2_ core, such as loss of a side-chain from an alkylated lactone ring. LiAlH_4_ reduction of the parent compound produced a number of minor products and one major product with the highest visible mass ion at *m/z* 182 (44% of base peak at *m/z* 121). This result was not immediately interpretable, other than indicating that there was at least one functional group such as a ketone or ester that was reducible by LiAlH_4_.

Because the extracts of volatiles consisted of essentially one compound, two of the extracts were combined, the methylene chloride solvent was replaced with deuteromethylene chloride, and the sample was analyzed by microprobe NMR. A 2-pentyl spin system was readily identified from a methyl triplet at 0.88 ppm (*J* = 7.2 Hz) coupled to a 2-proton methylene multiplet at 1.13–1.29 ppm, which in turn was coupled to two geminal diastereotopic protons at 1.61–1.71 (m) and 1.42–1.50 (m) respectively. This diastereotopic methylene group was coupled to a downfield-shifted proton at 2.75–2.84 (m), which was also coupled to a methyl group at 1.17 ppm (d, *J* = 6.8 Hz). The remaining protons consisted of a singlet methyl group at 1.94 ppm, and a methyl doublet at 1.99 ppm with a small ~1.5 Hz coupling to a proton at 6.95 ppm, suggestive of a 4-bond coupling between an allylic methyl group and an alkene proton. The two methyl groups and the 2-pentyl sidechain accounted for 7 of the 12 carbons but none of the sites of unsaturation, so the remaining 5 carbons and two oxygens had to be accommodated in a highly unsaturated structure. The fact that the molecule had been reduced by LiAlH_4_ suggested that at least one of the oxygens was probably present as a carbonyl, either in an unsaturated ring, or attached to an unsaturated ring as an acetyl group. This suggested either a 6-membered pyrone or 4H-pyran-4-one ring, or a 5-membered furan ring with an attached acetyl group. Spectral simulations, particularly of the carbon chemical shifts, suggested that a pyrone structure was most likely.

The substitution pattern on the pyrone ring was determined from consideration of the small 4-bond coupling between the methyl at 1.99 ppm and the alkene proton at 6.95 ppm, suggesting that they were attached to the same double bond. Furthermore, there were NOESY cross-peaks between the alkene proton and the two downfield methyl groups, indicating that the proton had to be on C3 with methyls on C2 and C4, or on C4 with methyls on C3 and C5. From biosynthetic considerations, the former structure seemed the most likely, with methyls on carbons 2, 4, and 6, as would be expected for a polypropanoate-based structure. This tentative structure was confirmed by synthesis. We have not yet determined which enantiomer the insect produces because the enantiomers (in the synthetic racemate) were not resolved on a chiral stationary phase Cyclodex B GC column.

### Synthesis of the male-produced compound

**Fig 5:**
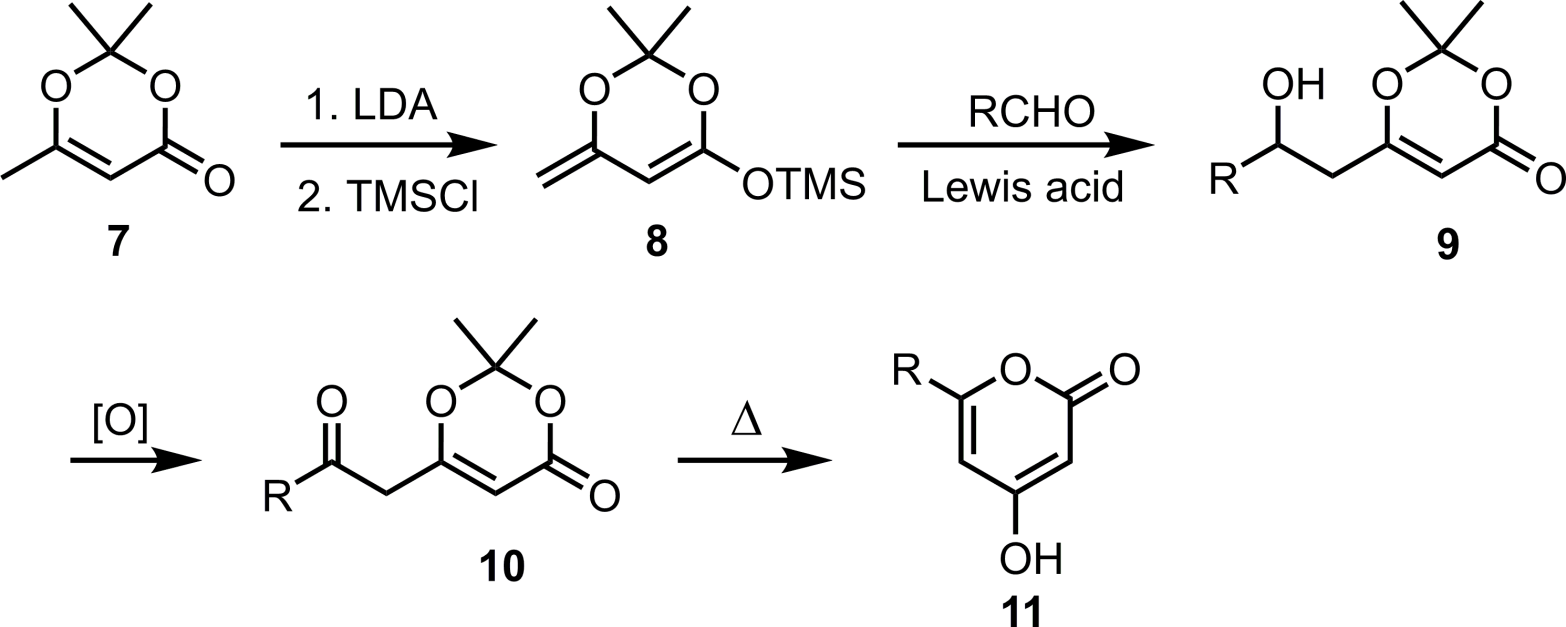
Multistep route from compound 7 to 10 and thermal cyclization to 11.

The synthesis of *Rosalia* pyrone **6** (Fig 2) started with 6-ethyl-2,2,5-trimethyl-1,3-dioxin-4-one **1**, which was readily prepared in two steps following the procedure reported by the Myers group [56]. In the past, the syntheses of 2,2-dimethyl-6-(2-oxyalkyl)-1,3-dioxin-4-one structures **10**, analogous to **3**, have been accomplished by a three step sequence of conversion of 2,2,6-trimethyl-1,3-dioxin-4-one **7** to its silyl ketene acetal derivative **8**, Lewis acid (BF_3_·Et_2_O, TiCl_4_, SiCl_4_ or MgBr_2_·Et_2_O) catalyzed vinylogous Mukaiyama aldol addition of **8** to aldehydes to give alcohols **9** [62–68], and oxidation (Fig 5).

However, the Katritzky group had reported a one-step conversion of **7** to **10** using 1-acylbenzotriazoles, and LDA as base [69, 70]. Although the reported yields were only modest for simple aliphatic 1-acylbenzotriazoles (ca. 50%), this methodology shortened the reaction sequence from 3 steps to one, and so was adopted to prepare the key intermediate **3**. Thus, nucleophilic acyl substitution of 1-acylbenzotriazole **2**, which was readily prepared from the corresponding carboxylic acid [55], with the carbanion generated from **1** provided **3** in 40% yield. As expected, **3** was formed as a mixture of *syn* and *anti* side chain 1,3-dimethyl diastereomers. The stereochemistry is ultimately of no consequence because the stereocenter at the 1 position of the side chain is eliminated in the next cyclization step (*vide infra*).

In the literature, a variety of 2,2-dimethyl-6-(2-oxoalkyl)-1,3-dioxin-4-ones **10** have been reported to undergo thermal cyclization smoothly to afford 6-substituted 4-hydroxy-2-pyrones **11** under conditions originally reported by Sato and coworkers (Fig 5), [71, 62, 65–67, 69, 70]. The reaction proceeds through an acyl ketene intermediate, which is generated by a retro-Diels-Alder reaction upon heating. Subsequent keto-enol equilibration and 6π-electron cyclization afforded the desired pyrone [71]. However, this transformation was not successful for **3**. When refluxed in toluene as reported in the literature, **3** was recovered quantitatively. Under harsher conditions (refluxing in xylenes), **3** was mostly recovered with only a trace amount of desired product **4** being observed by TLC. This result was initially inexplicable. Intermediate **3** has two extra methyl groups compared to **10**, the methyl group at the 1-position of the side chain and the 5-methyl group in the ring. Thermal cyclizations of similar substrates with alkyl groups (ethyl, *n*-butyl, and *n*-pentyl) at the 1-position of the side chain had been successful, giving 5-alkyl 6-substituted 4-hydroxy-2-pyrones [69]. Although there was no literature precedent for substrates with the 5-methyl group in the ring undergoing similar conversion (i.e., **10** to **11**), such substrates had been used as precursors of acyl ketenes in the formation of macrocyclic lactones [68, 72]. In addition, when **3** was subjected to GC-MS analysis (injector temperature 280°C), **3** was cleanly converted to **4** by a thermal cyclization, as evidenced by the single peak having the mass spectrum corresponding to **4**. Instead of further exploration of other thermal cyclization conditions, we tested an alternate route based on the sodium methoxide mediated cyclization of a very similar substrate reported by the Omura group [73], presumably via deprotection of the acetonide group and subsequent cyclization of the resulting diketoester. Gratifyingly, **3** was converted to pyrone **4** in good yield (76%) under these conditions.

**Fig 6:**
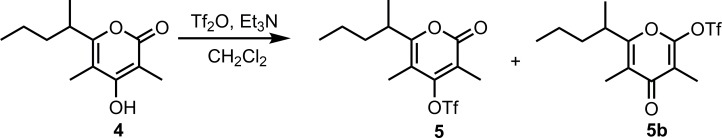
Triflation of 4 with triflic anhydride.

To achieve deoxygenation at C-4 of the pyrone ring, the hydroxypyrone **4** was first converted to triflate **5**. When the reaction was carried out with triflic anhydride and triethylamine in CH_2_Cl_2_ [74–76], the desired product **5** was formed along with a minor amount of its regioisomer **5b** [74], and the ratio of **5**/**5b** appeared to be dependent on the purity of the triflic anhydride (Fig 6). However, reaction of **4** with *N*-phenyl-bis(trifluoromethanesulfonimide) in the presence of potassium carbonate in THF at 60°C [77] proved to be much cleaner and more reliable, producing **5** as the only product in excellent yield (Fig 5). Finally, reduction of triflate **5** with triethylsilane and Pd catalysis gave deoxygenated target compound **6** in excellent yield [74, 76].

### Field bioassays of the synthesized compound

A total of 83 *R*. *alpina* males and females were captured during field bioassays at three study sites. The sex ratio of all captured beetles was equal (45 males and 38 females captured χ^2^ = 0.59, P = 0.44).

**Fig 7:**
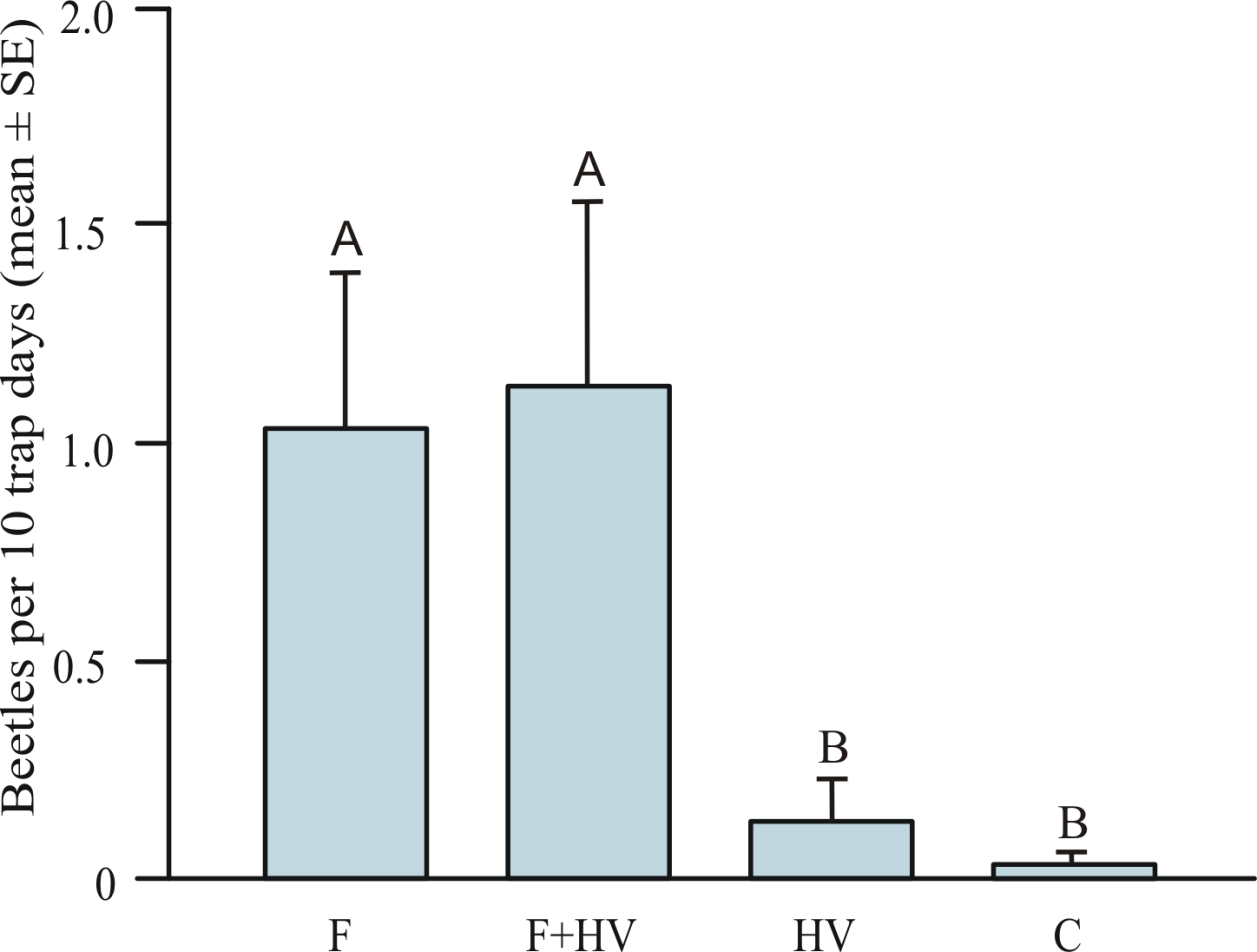
Relative abundance of beetles captured (mean ± SE) in traps baited with synthetic pheromone (F), pheromone + host volatiles (F + HV), host volatiles alone (HV), or controls (C) for three study sites in Slovenia (pooled data). Means significantly different: Kruskall-Wallis ANOVA H = 15.33, P < 0.01. Means with different letters are significantly different (Mann-Whitney test, Bonferroni corrected P < 0.05).

There were significant differences among treatments in the mean number of captured beetles (Kruskall-Wallis ANOVA H = 15.33, P < 0.01, Fig 7). Traps baited with the racemic synthetic pheromone (alone and in combination with host plant volatiles) consistently (i.e. at all three study sites and throughout the whole study period), captured more beetles (mean of 1.08 ± 0.22 beetles per 10 trap days; N = 77 beetles) than treatments without the pheromone compound (i.e. control traps, and traps baited with host plant volatiles; mean of 0.08 ± 0.04 beetles per 10 trap days; N = 6 beetles). The mean number of beetles attracted to the pheromone (1.03 ± 0.36 beetles per 10 trap days) was significantly different from the mean numbers attracted to host plant volatiles (0.13 ± 0.09 beetles per 10 trap days, Mann-Whitney U test, P < 0.01) or the control (0.03 ± 0.03 beetles per 10 trap days, Mann-Whitney U test, P < 0.01) (Fig 7). The host plant volatiles did not synergize attraction to the pheromone because there was no significant difference between the mean number of beetles attracted to the pheromone alone (1.03 ± 0.36 beetles per 10 trap days, Mann-Whitney U test, P = 0.89) compared to the pheromone in combination with host plant volatiles (mean of 1.14 ± 0.41 beetles per 10 trap days).

In addition, the number of captured beetles also differed significantly among the treatments within each of the individual study sites (Friedman’s 9.8308 < Q < 16.9182, 0.001 < P < 0.05). Traps baited with synthetic pheromone captured significantly more beetles than controls at all study sites (Dunn’s multicomparison test, 0.01 < P < 0.05) (Table 2). There was no significant difference in the number of captured beetles between the pheromone alone or the pheromone in combination with host plant volatiles treatments (Dunn’s multicomparison test P = 1.000 for all study sites).

**Table 2:** The number of beetles captured for different treatments within each of the individual study sites. The mean numbers captured per 10 trap days (±SE) are shown.

Treatment	Boč (mean ± SE)	Kum (mean ± SE)	Krim (mean ± SE)
pheromone (F)	1.16* ± 0.47	0.91* ± 0.61	1.03* ± 0.67
pheromone compound +	1.94** ± 0.43	0.71 ± 0.7	0.77* ± 0.44
host plant volatiles (F+HV)			
host plant volatiles (HV)	0.31 ± 0.21	0	0.08 ± 0.08
control (C)	0.07 ± 0.08	0	0

Significant differences between controls and pheromone treatments are indicated with asterisks (multicomparison Dunn’s test

*P < 0.05

**P < 0.01).

## Discussion

Analytical and field bioassay data suggest that the *Rosalia alpina* pheromone (alkylated pyrone) is a single male-produced compound that is attractive to both sexes. While checking the traps, we also observed strong behavioural effects, for example flying individuals, which, when they encountered the pheromone plume, responded by turning towards the pheromone baited-trap, landing on it, and orientating towards the lure. We also observed beetles being attracted and landing nearby while we were refilling the pheromone lure sachets.

*Rosalia alpina* males and females were attracted in approximately equal numbers, indicating that the pyrone is an aggregation pheromone. This is also the case with all other known pheromones produced sex-specifically by male cerambycids, including the North American congener *R*. *funebris* [50, 53]. In *R*. *funebris* the sex ratio of captured beetles was female-biased [53], while in *R*. *alpina* an equal sex ratio of captured beetles was observed. However, this data must be treated with caution, because the natural sex ratios of both species are not known. Also, captures of either sex could be influenced by factors such as seasonal and daily activity of the sexes, habitat preferences, asynchronous maturation, distribution patterns, and abiotic conditions (e.g., [78, 79]). To date the majority of pheromones identified from rare or endangered insect species in general have been female-produced sex pheromones, with only one other example of a male-produced aggregation pheromone, for the scarab beetle *Osmoderma eremita* (Scopoli), in which males attract both females and males to suitable habitats [80]. Whereas the function of aggregation behaviour in insects is very diverse, in the subfamily Cerambycinae the male-produced pheromones are considered to be particularly important in mating, to bring the sexes together [50, 81]. In addition to this role, the pheromone of *R*. *alpina* males may also help to aggregate both females and males at suitable host trees, where multiple females may oviposit. The aggregation habits of saproxylic beetles at suitable host trees have not been well studied, but can be rationalized on the basis of suitable dead wood substrates being a scarce resource, which can accommodate the offspring of multiple females, particularly if the larvae show a tolerance for close coexistence, as shown for example in cucujid and pyrrochroid species larvae [82].

We have not determined how much pheromone is actually released by individual males, but analyses of the aeration extracts showed that, as with many other species in the subfamily Cerambycinae, *R*. *alpina* males produce comparatively large amounts of pheromone (several tens of micrograms or more [50]), to the extent that two aeration extracts from males provided sufficient material for NMR analysis.

The structure of the *R*. *alpina* pheromone is not only a new natural product, but also the first example of an entirely novel structural class of pheromones within the Cerambycidae, being completely unlike that of any of the pheromones identified from cerambycid species to date. Also, very unexpectedly, the structure is completely unlike the pheromone ((*Z*)-3-decenyl (*E*)-2-hexenoate) of its North American congener, *R*. *funebris* [53]. However, other compounds containing the pyrone structural motif have been found in a wide variety of organisms, in which they have very diverse biological functions, including as metabolic intermediates and products, and signalling molecules mainly used in antagonistic interactions [83]. In particular, a pyrone compound has been reported as a sex pheromone component for the brown-banded cockroach (*Supella longipalpa*) [84], and recently pyrones have been shown to mediate communication in the bacterium *Photorhabdus luminescens* [85]. Further investigation of the other four species of the *Rosalia* genus or other members of its tribe (Compsocerini) would be helpful in determining whether pheromones may be unique to individual species in this group, or whether pheromone components are indeed generally shared among related species, as is often the case in other members of the subfamily Cerambycinae [86].

These were the first trials of the synthesized. *R*. *alpina* pheromone, with the principal goal of verifying whether or not the compound was attractive at all. Further investigation could improve the attractiveness of the pheromone, for example by optimizing the pheromone release rate. In this study we carried out tests using only one dose because of the limited amount of synthetic pheromone available. In addition, field trials used racemic synthetic pheromone because we have not yet determined which enantiomer the beetles produce, nor have we developed an enantioselective synthesis. Third, we used traps that were not originally designed for *R*. *alpina*, and modifications may improve trapping efficiency. In particular, while checking traps, we observed beetles walking on the vertical panes of the trap without falling down into the collection bucket, indicating that the traps were likely collecting only a fraction of the overall responders. It is known that the efficiency of trapping cerambycid beetles can be greatly enchanced by treating the traps with a lubricant, such as the fluoropolymer Fluon PTFE [87–90]. However, in our study we used non-coated traps because we were testing the efficacy of the pheromone of this highly endangered species for the first time. Thus, we were hesitant to use Fluon, which has been shown to increase the capture efficiency up to ten times, lest we substantially affect the beetle populations. Even without the Fluon treatment, trap captures were sufficient to show that the identified compound is indeed an aggregation pheromone for *R*. *alpina*. Having shown this, in ongoing trials we will test traps treated with Fluon to determine its effects on improving the trapping efficiency, and hence the monitoring, of this particular species.

The present study, along with several previously reported studies [11], has shown that pheromone-based trapping methods can increase the efficiency and cost-effectiveness of sampling rare and endangered species, and improve the accuracy and quality of target species population surveys and monitoring. Pheromone-based sampling is less liable to bias than most other sampling methods because it can be better standardized for weather conditions, spatial distribution, time of day, search/human effort, and other parameters at the national and international level, and applied in large-scale (spatially and temporally) surveys. For instance, the initial research effort and costs of identification and synthesis of the *R*. *alpina* pheromone were relatively high, but were a one-time cost. Based on established use of pheromone-baited traps in other systems, the time, labour, and costs of future sampling efforts can be significantly reduced compared to other commonly used methods. Finally, pheromone-based sampling of *R*. *alpina* can specifically facilitate its monitoring and conservation strategies in regions where numbers have already decreased significantly and the species is present in low densities, and consequently very difficult to detect by visual surveys. Drag and co-workers [91] also suggested that applying conservation measures over relatively large spatial scales is likely to be more efficient for conservation of highly mobile species such as *R*. *alpina*. We further suggest that application of pheromone-based sampling of *R*. *alpina* would provide much finer scale spatio-temporal data on the presence as well as the absence of this species, allowing for much more complete and reliable records of its distributions throughout Europe. However, identification and exploitation of pheromones of endangered species also has some potential risks [11], particularly for large and colourful species like *R*. *alpina*, which are sought after by collectors. Due to their efficiency in attracting the target species, uncontrolled use of pheromones for collecting specimens could further threaten already declining populations. Therefore the use and/or accessibility of pheromones of endangered species may require regulation. In addition, development of nonlethal trap designs is warranted, so that individuals can be counted in surveys but then released back into the population unharmed.

Finally, for some cerambycid species, attraction to the male-produced aggregation pheromones is strongly synergized by host plant volatiles [92–96]. In our study, we saw no evidence that the host plant volatiles (*Z*)-3-hexen-1-ol [95] and ethanol (the latter is specifically known to be a generic cerambycid attractant [96]), influenced attraction of *R*. *alpina* to the pheromone lures. However, more detailed study of the possible importance of host plant volatiles in attraction of this species is probably warranted, using blends that more closely resemble those emitted by their typical beech tree hosts, rather than very generic compounds or blends that are common to many plant species.

## Conclusions

The present study showed that male *R*. *alpina* produce comparatively large amounts of a novel, sex-specific compound which acts as an aggregation pheromone. Whereas fine-tuning the pheromone release rate and trap design should improve trapping efficiency, our preliminary field bioassays have already provided strong evidence that the *R*. *alpina* pheromone can be used to significantly enhance survey methods for this species, particularly as only direct observation methods are currently used, such as daytime surveys of adult beetles, counting emergence holes in host trees, or observing attraction of beetles to piles of host logs [34, 43, 46, 91]. These observation methods are very labour intensive, of relatively low efficiency, and are strongly dependent on the beetle’s daytime activity level. Moreover, the pheromone could be a useful tool for developing a much more detailed understanding of the biology and ecology of this species, including its activity and dispersal patterns, reproductive biology, and habitat preferences, all of which would harmonize conservation issues with forest exploitation and facilitate land-management decisions of protected and unprotected areas.
